# Recent Advances in Ocular Drug Delivery: Insights into Lyotropic Liquid Crystals

**DOI:** 10.3390/ph17101315

**Published:** 2024-10-02

**Authors:** Samer Adwan, Madeiha Qasmieh, Faisal Al-Akayleh, Ahmed Saad Abdulbari Ali Agha

**Affiliations:** 1 Department of Pharmaceutics and Pharmaceutical Technology, Faculty of Pharmacy, Zarqa University, Zarqa 13110, Jordan; 20160132@zu.edu.jo; 2 Department of Pharmaceutics and Pharmaceutical Technology, Faculty of Pharmacy and Medical Sciences, Petra University, Amman 11196, Jordan; falakayleh@uop.edu.jo; 3 School of Pharmacy, Department of Pharmaceutical Sciences, The University of Jordan, Amman 11196, Jordan; asat3u@gmail.com

**Keywords:** ocular drug delivery, lyotropic liquid crystals, liquid crystal nanoparticles, cubosomes, bulk forming, machine learning, biosensing applications

## Abstract

**Background/Objectives:** This review examines the evolution of lyotropic liquid crystals (LLCs) in ocular drug delivery, focusing on their ability to address the challenges associated with traditional ophthalmic formulations. This study aims to underscore the enhanced bioavailability, prolonged retention, and controlled release properties of LLCs that significantly improve therapeutic outcomes. **Methods:** This review synthesizes data from various studies on both bulk-forming LLCs and liquid crystal nanoparticles (LCNPs). It also considers advanced analytical techniques, including the use of machine learning and AI-driven predictive modeling, to forecast the phase behavior and molecular structuring of LLC systems. Emerging technologies in biosensing and real-time diagnostics are discussed to illustrate the broader applicability of LLCs in ocular health. **Results:** LLCs are identified as pivotal in promoting targeted drug delivery across different regions of the eye, with specific emphasis on the tailored optimization of LCNPs. This review highlights principal categories of LLCs used in ocular applications, each facilitating unique interactions with physiological systems to enhance drug efficacy and safety. Additionally, novel applications in biosensing demonstrate LLCs’ capacity to improve diagnostic processes. **Conclusions:** Lyotropic liquid crystals offer transformative potential in ocular drug delivery by overcoming significant limitations of conventional delivery methods. The integration of predictive technologies and biosensing applications further enriches the utility of LLCs, indicating a promising future for their use in clinical settings. This review points to continued advancements and encourages further research in LLC technology to maximize its therapeutic benefits.

## 1. Introduction

**Figure 1:**
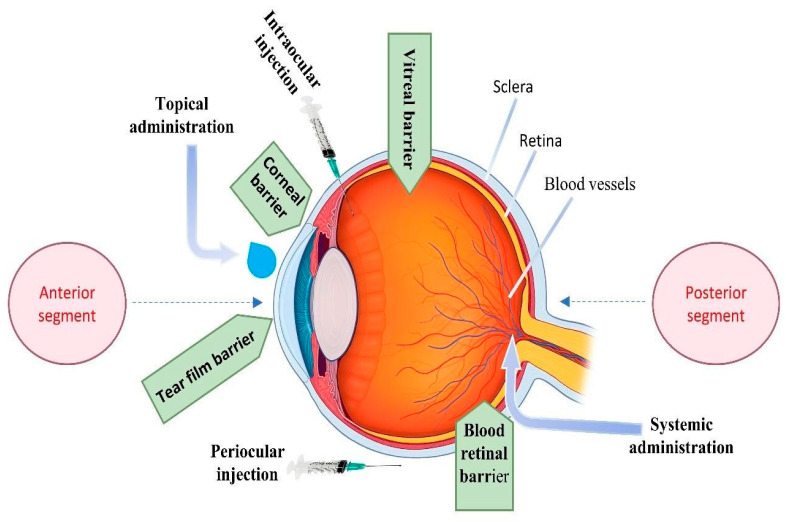
A schematic illustrating the anatomical regions of the eye, the physiological barriers impeding ocular drug delivery, and the various methods employed for ocular drug administration.

The World Health Organization estimates that at least 2.2 billion people suffer from visual impairment globally, with nearly half of these cases being preventable or undertreated. This burden is exacerbated by limitations in accessibility and quality of vision care, particularly in low-income and middle-income countries [1]. Despite the prevalence of these conditions, the efficient delivery of drugs to ocular tissues remains a significant challenge due to the eye’s complex anatomy and protective barriers, as shown in Figure 1. Key barriers include the tear film, which rapidly clears topical drugs; the corneal barrier with tight junctions that limit drug penetration; the vitreal barrier, where the gel-like vitreous humor hinders drug diffusion to the posterior segment; and the blood–retinal barrier (BRB), which restricts systemic drugs from reaching retinal tissues [2]. These barriers collectively reduce the effectiveness of ocular drug delivery, necessitating advanced strategies to enhance therapeutic outcomes [3]. These barriers, along with others listed in Table 1, significantly reduce the bioavailability of conventional ophthalmic formulations to typically less than 5%. This low bioavailability necessitates frequent dosing, which often results in reduced patient compliance [4].

To address these limitations, there has been a growing interest in advanced drug delivery systems, particularly lyotropic liquid crystals (LLCs). LLCs are self-assembled structures formed when amphiphilic molecules, such as lipids or surfactants, organize in the presence of a solvent, typically water. LLCs exhibit a unique mesophase that combines the ordered structure of crystalline solids with the fluidity of liquids, making them highly suitable for drug delivery applications [15].

Their ability to self-assemble into various mesophases, including lamellar, hexagonal, and cubic structures, allows for the encapsulation and controlled release of a diverse array of therapeutic agents [16]. These properties are particularly advantageous for ocular drug delivery, where sustained release and prolonged retention time are critical for enhancing therapeutic efficacy [17].

Recent advancements have expanded the application of LLCs beyond traditional pharmaceutical uses. The integration of predictive modeling, enhanced by machine learning (ML) techniques, has revolutionized the design and optimization of LLC-based drug delivery systems [18]. This approach allows for more accurate predictions of phase behavior and molecular ordering, thereby reducing the need for extensive experimental trials and accelerating the development of optimized formulations [19]. Additionally, LLCs have shown significant promise in biosensing, where their unique optical properties, coupled with AI-driven analyses, have markedly improved the sensitivity and specificity of biosensors used in medical diagnostics, environmental monitoring, and food safety [20,21].

Despite these advancements, there remains a lack of comprehensive reviews focusing on integrating LLCs in ocular drug delivery, particularly those incorporating the latest developments in predictive modeling and biosensing technologies. This review uniquely addresses this gap by providing an in-depth analysis of recent progress in LLC-based ocular drug delivery systems, highlighting how advanced techniques like machine learning and AI-driven predictive modeling enhance the design and optimization of these systems. By exploring emerging applications beyond traditional methods, this review offers novel insights into the transformative potential of LLCs in improving therapeutic outcomes for ocular diseases.

This review aims to provide a comprehensive examination of recent advancements in LLC technology, with a particular emphasis on their application in ocular drug delivery. It explores the potential of both bulk-forming LLCs and liquid crystal nanoparticles (LCNPs), highlighting innovative approaches that have emerged in recent years. Additionally, this review addresses novel methodologies extending beyond traditional LLC techniques, such as predictive modeling, an automated analysis, and enhanced biosensing capabilities, which have significantly optimized drug delivery systems. By analyzing these developments, this review aims to offer insights into the potential of LLC-based therapies to improve therapeutic outcomes for patients with ocular diseases and other related conditions.

## 2. Ocular Administration Routes

Given the unique physiological barriers of the eye, various administration routes have been developed to optimize drug delivery and therapeutic efficacy [22]. Each route offers distinct advantages and limitations, depending on the targeted ocular segment and the type of disease being treated [22]. Table 2 provides an overview of these administration routes, highlighting their mechanisms, applications, and associated challenges.

## 3. Advancements in Ocular Drug Delivery Systems

**Figure 2:**
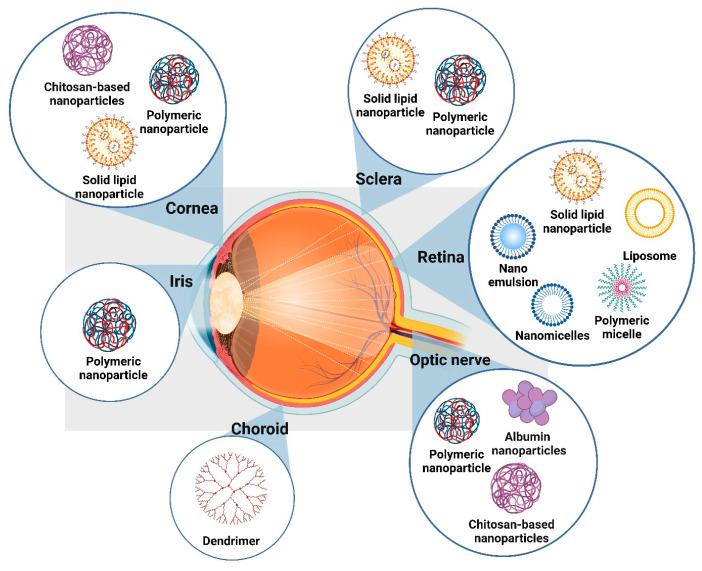
Nanoparticle-based systems for targeted ocular drug delivery across various eye regions.

Over the years, significant advancements have been made in ocular drug delivery systems, addressing the limitations of traditional methods [31]. Innovations such as drug-loaded contact lenses, punctum plugs, implants, microneedles, and in situ gelling systems have expanded the possibilities for sustained and targeted drug delivery, improving therapeutic outcomes [31]. Alongside these developments, nanoparticle-based systems have emerged as a crucial tool in enhancing ocular drug delivery [32]. Nanoparticles, including nanomicelles [33], polymeric nanoparticles [34], and cubosomes [35], offer unique benefits, such as the ability to cross ocular barriers, improve drug solubility, and provide controlled release, thereby increasing bioavailability and therapeutic efficacy [35]. Recent advancements in nanoparticle-based drug delivery systems have further enhanced the therapeutic potential in ocular applications by improving drug bioavailability, targeting, and sustained release across various eye regions. Figure 2 provides a comprehensive overview of these nanoparticle systems and their targeted delivery sites within the ocular anatomy.

Chitosan-based nanoparticles, with their mucoadhesive properties, enhance drug retention on the corneal surface and the optic nerve by facilitating binding with negatively charged ocular tissues [36]. Polymeric nanoparticles are particularly versatile, targeting the cornea, iris, sclera, and optic nerve while offering controlled and sustained drug release [37]. Solid lipid nanoparticles (SLNs), used in corneal, scleral, and retinal applications, improve drug stability and provide prolonged release [38]. Liposomes are effective in retinal drug delivery, where their phospholipid bilayer allows for direct drug fusion with retinal cells [39]. Nanoemulsions and nanomicelles increase the solubility and bioavailability of hydrophobic drugs, ensuring sustained release to the retina [40,41]. Dendrimers, due to their high drug-loading capacity and targeted delivery, are suitable for applications targeting deep ocular tissues like the choroid [42]. Additionally, albumin nanoparticles demonstrate effective crossing of the blood–retinal barrier, enhancing drug delivery to the optic nerve and posterior ocular structures [43]. These advancements underscore the potential of nanoparticle-based systems to overcome traditional challenges in ocular drug delivery, offering new avenues for targeted and sustained treatment of various eye conditions.

These advancements are also shown in Table 3, which details both the innovative ocular drug delivery systems and the various nanoparticle-based approaches, along with their mechanisms and the potential benefits they offer for improving ocular drug delivery.

## 4. Composition, Formation, and Applications of Lyotropic Liquid Crystals in Drug Delivery

Recent progress in pharmaceutical formulations has brought attention to the possibility of LCs’ dosage forms for delivering drugs that are soluble in both water and oil. LCs possess a unique mesophase that allows them to exhibit properties that are characteristic of both crystalline solids and genuine liquids. Their ability to both maintain a structured arrangement and demonstrate fluidity makes them highly beneficial for pharmacological applications [56]. The mesophase of LCs is an intermediate state that combines the structural order of solid phases with the fluidity of liquid phases [57]. This state plays a crucial role in pharmaceutical sciences as it enables the accurate arrangement and positioning of molecules, which is essential for the controlled release and targeted distribution of medications [58]. In addition, LCs have the ability to spontaneously arrange themselves into intricate and enduring formations that exhibit a high degree of organization across one, two, or three dimensions [59]. The structural arrangement of medications is not only a physical characteristic but also has a significant impact on improving their pharmacokinetic qualities [56]. The structured and adaptable nature of these formations facilitates the efficient dispersion of active medicinal ingredients, leading to enhanced solubility, improved stability, and optimized bioavailability of the pharmaceuticals [60].

**Figure 3:**
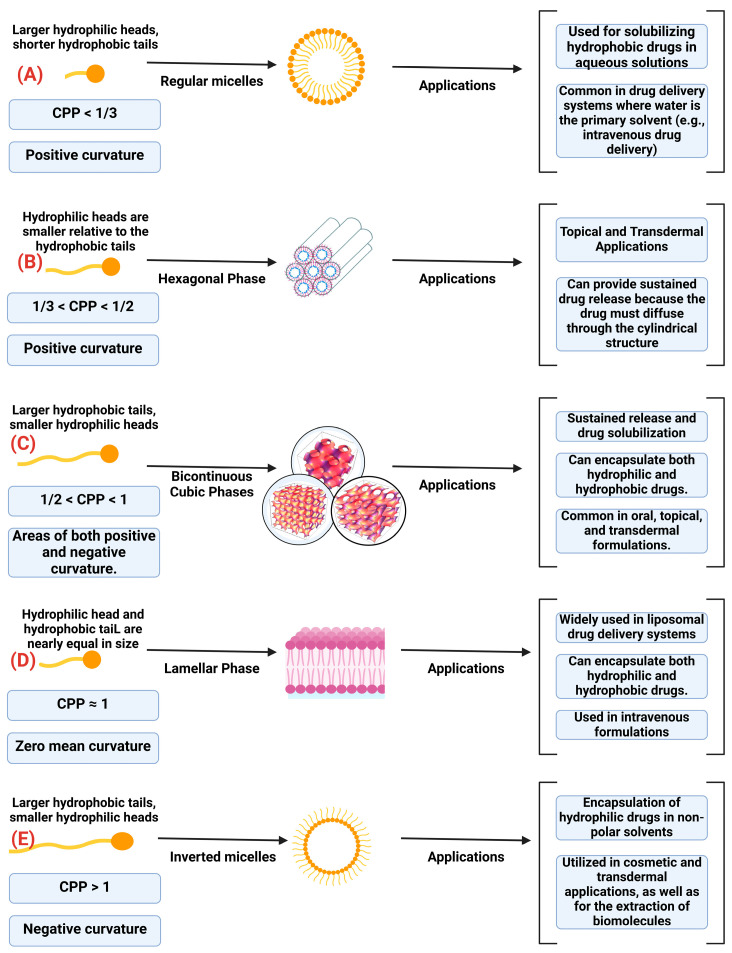
Schematic representation of lyotropic liquid crystal phases and their pharmaceutical applications.

Understanding the self-assembly of amphiphilic molecules into LLC phases is crucial for designing effective drug delivery systems. This self-assembly is governed by the critical-packing parameter (CPP), defined as Equation (1), where *v* is the hydrophobic tail volume, *a* is the hydrophilic head area, and *l* is the tail length. The CPP dictates the curvature and resulting structure of the aggregates formed. Figure 3 illustrates how varying CPP values lead to the formation of specific LLC phases—regular micelles, hexagonal phases, bicontinuous cubic phases, lamellar phases, and inverted micelles—and their corresponding pharmaceutical applications.

*CPP* = *v*/(*a* ∗ *l*) (1)

When the CPP is less than 1/3, the amphiphilic molecules arrange into spherical micelles due to large hydrophilic head groups and short hydrophobic tails, resulting in high positive curvature (Figure 3A) [61]. These regular micelles are particularly effective in solubilizing hydrophobic drugs within aqueous environments, enhancing their bioavailability in intravenous formulations. As the CPP increases to between 1/3 and 1/2, the curvature decreases, and the molecules assemble into cylindrical micelles that organize into a hexagonal phase (Figure 3B) [61]. Here, the hydrophilic heads are smaller relative to the longer hydrophobic tails. The hexagonal phase offers enhanced structural stability, making it advantageous for topical and transdermal applications where controlled and sustained drug release is desired. When the CPP ranges from 1/2 to 1, the molecules form bicontinuous cubic phases characterized by complex, interconnected networks of lipid bilayers and water channels exhibiting both positive and negative curvature (Figure 3C) [62]. These structures arise due to significantly larger hydrophobic tails than hydrophilic heads. Bicontinuous cubic phases are highly versatile in pharmaceutical formulations and can encapsulate both hydrophilic and hydrophobic drugs. They are suitable for oral, topical, and transdermal delivery systems that require sustained release profiles. At a CPP of approximately 1, the amphiphilic molecules arrange into lamellar phases consisting of flat bilayers with zero mean curvature (Figure 3D) [62]. This structure results from the nearly equal sizes of the hydrophilic head and hydrophobic tail. Lamellar phases form the basis of liposomal drug delivery systems, which can encapsulate a broad spectrum of therapeutic agents. These systems enhance stability and bioavailability, particularly in intravenous applications. When the CPP exceeds 1, the curvature becomes negative, leading to the formation of inverted micelles (Figure 3E) [63]. In this phase, hydrophilic heads are oriented inward, and hydrophobic tails face outward into a non-polar solvent. This inverted structure is particularly useful for encapsulating hydrophilic drugs within lipid-based or oil-based formulations. Such formulations are commonly employed in cosmetic products, transdermal delivery systems, and biotechnological applications for biomolecule extraction. By comparing these structures, we observe a gradual transition from high positive curvature to negative curvature as the CPP increases. This transition is accompanied by changes in the self-assembled morphology, directly impacting the pharmaceutical applications of each LLC phase. By strategically manipulating the CPP through molecular design, altering head group size, tail length, or tail volume, scientists can tailor LLC structures to optimize drug delivery efficiency, stability, and controlled release mechanisms.

Within the realm of drug delivery devices, LCs can be categorized into two primary types, thermotropic and lyotropic. Thermotropic LCs arise from the self-assembly of pure drug molecules at different temperatures without the use of a solvent [64]. This specific type of LC is especially advantageous for medications that necessitate accurate heat regulation in order to enable effective administration at the intended location [65]. LLCs are formed when amphipathic lipids or surfactants interact with water. These systems depend on the creation of micelles or comparable structures through the addition of a surfactant to a solvent, usually water. LLCs are adaptable for creating aqueous drug delivery systems that may effectively transport pharmaceuticals with different solubilities, such as hydrophilic, hydrophobic, or amphiphilic medications [65]. Both categories of LCs present unique benefits in drug formulation, offering a flexible foundation for improving drug stability and delivery. As ongoing research progresses, LCs are increasingly recognized for their potential to revolutionize pharmaceutical development by enabling the development of more efficient and precise drug delivery systems.

## 5. Classification of Lyotropic Liquid Crystals

**Figure 4:**
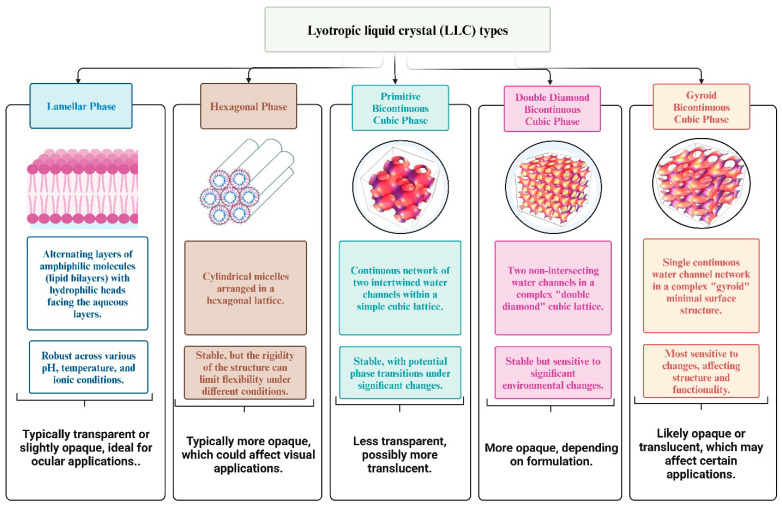
Key types of LLCs and their structural characteristics relevant to ocular applications.

Recent advancements have highlighted the important function of LLCs in improving the delivery of medication through mucosal surfaces. These systems are highly appreciated for their exceptional capacity to efficiently penetrate biological barriers and sustain a long-term presence at the site of administration, which has the potential to enhance therapeutic effectiveness [66]. LLCs are created through the manipulation of the concentration of specific substances, such as surfactants, within a specific range of temperatures. As the solute concentration increases, distinct mesophases become apparent, indicating varied structural and functional properties [67]. The mesophases, namely the lamellar, hexagonal, and cubic phases shown in Figure 4, result from the self-organization of amphiphilic lipids due to variations in temperature and composition.

The self-assembly process is primarily driven by hydrophobic interactions that take place when these lipids are exposed to water, resulting in the formation of organized aggregates [68]. The reverse cubic and reverse hexagonal phases are highly suitable as delivery systems for a wide range of medicinal applications among the LLCs [69]. The internal composition of these stages enables the inclusion and secure containment of different therapeutic molecules, including those that are water-loving, fat-loving, and both water-loving and fat-loving in nature [70]. This provides possibilities for precise and regulated release mechanisms [71]. The cubic phase LC structures display a distinct duality in their structural formation. They can either appear as typical micellar aggregates with continuous water channels and fragmented hydrocarbon regions or as reversed aggregates with the opposite configuration [72]. Their ability to adapt structurally contributes to their ability to control the release of drugs. In addition, cubic phase LLCs exhibit notable mechanical rigidity in comparison to lamellar and hexagonal LCs, thereby impeding the unregulated movement observed in these mesophases [73]. When there is too much water added, the cubic phase changes into a clear gel that is thick and stretchy [74]. This gel is stable and has the same properties in all directions. These gels, although stiff, are difficult to detect using conventional polarized electron microscopy, emphasizing their distinctive physical properties [60]. The hexagonal phase is distinguished by the arrangement of cylindrical micelles into a three-dimensional hexagonal lattice. When exposed to organic solvents, this structure can undergo reversal, resulting in the formation of the reverse hexagonal phase. When adequately mixed with water, these hexagonal structures exhibit a unique fan-like texture that is visible when observed under polarized light microscopy [58]. Ultimately, the lamellar LCs, also known as neat phases, expand significantly, creating substantial structures resembling sheets that are interspersed with channels of water. The presence of a bilayer structure in these phases reduces the interaction between the oil and aqueous phases, which is advantageous for the encapsulation and preservation of drugs. The arrangement can be observed using polarized light microscopy, which reveals a unique streaky or mosaic-like texture, indicating its anisotropic characteristics [60].

While texture observations through polarized light microscopy can provide valuable insights into the structural organization of LLCs, the definitive determination of the internal structure requires the use of small-angle X-ray scattering (SAXS) or diffraction techniques [75]. These methods are essential for accurately characterizing the mesophase structures, including identifying lattice parameters and confirming the presence of specific phases such as lamellar, hexagonal, or cubic [76].

Aside from their structural adaptability, LLCs demonstrate a range of physiological impacts beyond their drug administration function. The effects are observed in several physiological systems, enhancing their therapeutic capacity in diverse applications [77].

**Figure 5:**
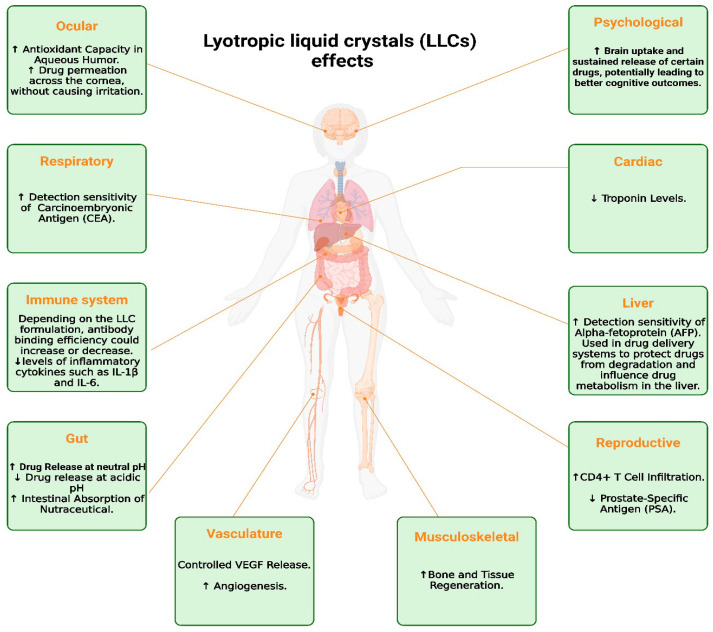
An overview of the systemic effects of LLCs across various organs and systems, highlighting their potential therapeutic applications. The arrows are used to indicate increases (upward arrows) and decreases (downward arrows) in specific physiological responses when using lyotropic liquid crystals (LLCs).

Figure 5 depicts the diverse effects of LLCs on different physiological systems, emphasizing their possible impact on eye health, immune system activities, and cardiovascular stability, among other related areas.

LLCs for ocular drug delivery are categorized into two main types based on their formulation strategy, bulk-forming liquid crystals and nanoparticle liquid crystals (LCNPs). Bulk-forming liquid crystals are extensively studied for their capacity to create sustained release systems. These bulk phases, including hexagonal and cubic structures, are engineered to maintain therapeutic levels of drugs over extended durations [78], whether applied locally or intravitreally, thereby enhancing the treatment efficacy for various ocular conditions. Conversely, liquid crystal-based nanoparticles, such as cubosomes, represent a significant advancement in ocular drug delivery by improving drug targeting, controlled release, and bioavailability [79]. Formulated with amphiphilic lipids, these nanoparticles effectively deliver therapeutic agents to the eye, overcoming traditional barriers and ensuring sustained therapeutic effects. Both bulk-forming and LCNPs exemplify innovative approaches in ocular drug delivery, offering substantial improvements in treatment outcomes for a range of eye diseases.

### 5.1. Bulk-Forming Liquid Crystals for Ocular Drug Delivery

Glycerol monooleate (GMO) is currently a highly researched amphiphilic lipid due to its potential use in the development of LLC medication formulations [80]. GMO, which is included in the FDA Inactive Ingredients Guide, is known for its non-toxic, biodegradable, and biocompatible properties. It is classified as Generally Recognized as Safe (GRAS) [81]. This approval highlights the potential appropriateness of using it in pharmaceutical applications, specifically for delivering therapeutics like vancomycin, as investigated by Milak et al. (2019) [68]. Milak et al. conducted extensive research on the utilization of GMO to create bulk hexagonal and cubic phases that are specifically tailored for ocular drug delivery systems [68]. These systems are designed to maintain therapeutic levels of Vancomycin HCl either locally in the eye or intravitreally for long periods of time. Their innovative method utilized melted homogenization and solvent evaporation techniques to create these phases, which effectively regulated the release rate of Vancomycin HCl [68]. The hexagonal phase, characterized by closed water channels, was found to considerably delay the release of Vancomycin HCl in comparison to the reverse cubic phase, which has open water channels [68]. Milak et al. conducted additional research in 2020 to examine the structural and release properties of liquid crystalline phases made from GMO/Paraffin oil/Vancomycin HCl. The liquid crystalline phases were altered by incorporating additives like polyglycerol ester or a triblock copolymer to assess and improve their capacity to sustain therapeutic Vancomycin HCl concentrations over prolonged durations [82], whether applied topically or injected intravitreally.

The objective of the study was to enhance the compositions for long-term treatment of eye infections. The results demonstrated encouraging prospects for both localized and systemic ocular applications [82].

Phytantriol (PHY) is another notable example of amphiphilic lipids that has been recognized for its non-toxic, mucoadhesive, and biocompatible characteristics. It is commonly used as a secure and efficient framework in LLC formulations [83]. Wang et al. (2019) utilized PHY to create a reversed bicontinuous cubic phase carrier for the transportation of pilocarpine nitrate, a medication frequently employed in the treatment of glaucoma [83]. The team aimed to reduce ocular irritancy and improve the bioavailability of pilocarpine nitrate by using a vortex method to mix PHY and water in a specific ratio. The LC gels produced were evaluated against conventional eye drops and exhibited a sustained release pattern, reduced corneal irritation, and prolonged drug retention in the ocular environment. These findings indicate a substantial enhancement over conventional delivery techniques [83]. Building upon this study, Xingqi et al. further examined the capability of PHY-based LC gels containing cubic and hexagonal phases for delivering pilocarpine nitrate to the eye [71]. Their research showed that these gels can effectively keep therapeutic levels of pilocarpine nitrate in the liquid part of the eye for at least 12 h after being given, which significantly improves the drug’s ability to be absorbed by the body and offers a more efficient treatment for glaucoma [71]. Additionally, Wu et al. created a novel in situ LC gel specifically formulated for the ophthalmic administration of dexamethasone [84]. This system was specifically designed to improve the retention of dexamethasone in the eye, resulting in higher levels of dexamethasone available in the eye and proving to be especially effective in treating DR [84]. Their research confirmed that the in situ-formed liquid crystal gel (ISLG) can release substances over an extended period, is highly compatible with living organisms, and is safe. This positions the ISLG as a promising foundation for future treatment approaches in managing ocular diseases [84]. Li et al. developed a new type of gel called resveratrol-loaded ocular lamellar crystalline gel (ROLG) to treat corneal neovascularization [66]. The research team employed advanced imaging techniques, such as polarized light microscopy and small-angle X-ray scattering, to confirm the presence of lamellar crystalline structures in the ROLGs. These gels exhibited strong retention properties on the surface of the eye, outstanding capacity to load drugs, and improved drug permeability through the tissues of the cornea. In addition, the gels were recognized for their simplicity of application and ability to provide a sustained release, making them an optimal vehicle for delivering resveratrol and a potentially effective new treatment option for managing corneal neovascularization [66]. Also, Tarsitano et al. investigated the use of LLCs as a novel ocular drug delivery system for the delivery of Acyclovir [85]. The study successfully demonstrated the use of a lamellar phase LC that transitions to a cubic phase in situ on the corneal surface, allowing for controlled drug release while overcoming issues with the physicochemical properties and limited precorneal retention time of many ophthalmic medications. The cubic phase exhibited constant viscosity and degradation kinetics across a variety of circumstances, indicating a significant potential for improving drug delivery efficacy. Ex vivo investigations on pig eyeballs and isolated corneas further proved the system’s stability and safety, with no deleterious alterations identified in corneal tissue structures [85]. The wide range of research highlights the powerful ability of LLCs to transform ocular drug delivery. Table 4 provides a summary of bulk-forming LLCs utilized in ocular drug delivery.

### 5.2. Liquid Crystal-Based Drug Nanoparticles (LCNPs) for Ocular Drug Delivery

The LCNPs are nanoscale particles that combine the ordered structure of crystals with the fluidity of liquids [86]. Formed by the self-assembly of amphiphilic molecules in solvents, LCNPs can encapsulate both hydrophilic and hydrophobic drugs, offering controlled release and enhanced bioavailability for drug delivery applications [86].

The application of LCNPs in ocular drug delivery has made remarkable progress, demonstrating improvements in therapeutic targeting, controlled and sustained release, stability, bioavailability, and the solubility of poorly soluble drugs. Research in this field over the past decade has established a strong foundation for ongoing and future innovations, confirming LCNPs as a transformative approach in ocular pharmacotherapy.

In the presence of water, lamellar phase LC structures commonly produce liposomes, which are spherical vesicles made up of one or more lipid bilayers. On the other hand, cubic and hexagonal phases can retain their unique geometric structures, resulting in the formation of cubosomes and hexosomes, which are nanoparticles with a well-defined internal architecture that is suitable for encapsulating and releasing drugs [67]. A pioneering study by Gan, Li et al. marked the inception of self-assembled LCNPs (cubosomes) for ocular dexamethasone delivery, demonstrating significantly enhanced aqueous humor pharmacokinetics compared to conventional eye drops. The study revealed that low-viscosity cubosomes containing 10% oil remained in the preocular region longer, leading to an eight-fold increase in the area under the curve (AUC) for dexamethasone compared to standard formulations [35].

Building on this foundation, Chen et al. (2012) developed LCNPs for cyclosporine A delivery, utilizing a combination of GMO and poloxamer 407 [81]. This formulation markedly improved corneal penetration and retention, surpassing the performance of oil solutions and offering a promising approach for treating ocular conditions with reduced irritation.

Similarly, Li et al. (2013) investigated LCNPs for pilocarpine nitrate delivery in glaucoma treatment. Their formulation, characterized by uniformly dispersed nano-sized particles, exhibited minimal ocular irritation, enhanced bioavailability, and sustained intraocular pressure reduction, presenting a significant advancement over commercial eye drops [87].

Further refining the application of LCNPs, Achouri et al. (2015) employed a design of experiment methodology to optimize nanoparticle formulations for keratoconus treatment. Their research identified critical parameters such as temperature, emulsification length, and homogenization, which are essential for achieving optimal particle size and drug encapsulation efficiency [88].

Hartnett et al. (2015) highlighted the stability of cubosomes derived from amphiphiles like PHY and GMO, which are well suited for prolonged drug release [89]. This stability is crucial for maintaining consistent therapeutic levels in treatments that require sustained medication release.

In 2016, Liu et al. introduced an innovative ocular delivery method using tetrandrine-loaded LCNPs [90]. This formulation demonstrated significantly improved ocular bioavailability and pharmacological benefits, suggesting a novel approach for enhancing drug retention and efficacy in eye treatments. Concurrently, Verma and Ahuja (2016) evaluated cubic LCNPs for tropicamide delivery, finding that these nanoparticles provided a faster onset of action and increased potency, offering potential improvements in eye care procedures [91].

Ali et al. (2016) developed a cubosomal system for ketorolac delivery, which improved transcorneal permeability and retention, highlighting the potential of cubosomes as a more effective alternative to traditional eye drops [92]. Younes et al. (2018) further advanced this field by creating a cubosomal delivery system for sertaconazole nitrate, enhancing the drug’s permeability and effectiveness in treating fungal keratitis—a significant challenge in ocular pharmacotherapy [93].

Recent studies have continued to push the boundaries of LCNP applications. Silva et al. (2019) designed a pirfenidone-loaded LCNP system that accelerated corneal healing, demonstrating the potential of LCNPs in treating corneal injuries [94]. Eldeeb et al. (2019) explored brimonidine tartarate-loaded cubosomes, which significantly improved the efficacy and permeability of the drug, offering a more effective and long-lasting treatment for glaucoma [95]. The promise of cubosomes in the treatment of glaucoma was highlighted by the findings of the study, which showed a 4.6-fold increase in AUC and a 1.6-fold enhancement in permeability in comparison to any commercially available products [95].

El-Gendy et al. (2020) investigated the inclusion of penetration enhancers in cubosome formulations, finding that these customized liquid crystalline nanostructures enhanced ocular drug delivery without causing irritation, thereby expanding their potential applications [96]. Kaul et al. (2021, 2022) studied the use of LCNPs for delivering tobramycin and vancomycin, showing improvements in preocular residence time and drug permeability, which could reduce dosing frequency and enhance treatment efficacy [97,98].

In 2021, Bessone et al. advocated for the use of cubic LCs for latanoprost delivery, demonstrating their ability to release the drug continuously over an extended period, potentially revolutionizing glaucoma treatment by reducing dosing frequency and limiting adverse effects [99]. Elfaky et al. (2021) developed a ketoconazole cubosomal gel that enhanced drug penetration and retention while displaying strong antifungal activity, highlighting the therapeutic potential of cubosomal formulations [100].

Recent advancements include Shoman et al.’s (2023) exploration of hyaluronan-based cubosomes loaded with bromfenac sodium, which improved corneal permeability and retention, potentially enhancing treatment outcomes for pterygium and cataract [101]. Nasr et al. (2023) revealed that fluconazole combined with cubosomal nanoparticles exhibited superior penetration and safety profiles, offering a more efficient topical therapy for keratomycosis [102]. According to the findings of both the ex vivo and in vivo trials, the corneal penetration was significantly higher, and the therapeutic effects were superior to those of aqueous solutions [102].

Further innovations in 2023 by El-Gendy et al. focused on improving the ocular bioavailability and therapeutic efficacy of Travoprost using liquid crystalline nanostructures [103]. Priya et al. (2023) developed a Loteprednol Etabonate-loaded LCNP gel, which demonstrated improved ocular retention and efficacy, marking a significant advancement in treating inflammatory eye conditions [104]. Similarly, Malaekeh-Nikouei et al. (2023) produced fluorometholone-loaded cubosomes, providing insights into efficient steroid delivery for ocular inflammation [105]. The formulation that was optimized did not exhibit any major changes in terms of physical characterization or in vitro release, which is evidence that the formulation showed perfect stability.

In a study by Sharadha et al. (2023), triamcinolone-loaded cubic LCNPs outperformed typical suspensions in drug delivery and therapeutic outcomes, suggesting the potential for improved nanocarriers for retinal therapeutics [106]. Omran et al. (2024) introduced a phytocubosomal system coated with chitosan and loaded with luteolin, demonstrating significant success in reducing intraocular pressure and inflammation in glaucoma treatment [107]. Those phytocubosomes exhibited sustained drug release, enhanced antioxidant activity, and increased ex vivo transcorneal penetration in comparison to the luteolin suspension.

Iyer et al. (2024) investigated an extended release cubogel formulation of moxifloxacin hydrochloride, showing potential for continuous release and enhanced bioavailability, positioning it as a promising alternative to traditional eye drops [108]. Chakorkar et al. (2024) optimized fluorometholone-loaded cubosomal vesicles using a quality by design approach, demonstrating improved drug release and ocular bioavailability [109]. Aher et al. (2024) examined acetazolamide-loaded cubosomes, highlighting enhanced corneal penetration and prolonged drug release, which could improve glaucoma treatment efficacy and patient compliance [110].

Finally, Nemr and Adel (2024) developed a fenticonazole-loaded cubosomal formulation that significantly improved corneal absorption and permeation, offering a transformative approach to treating fungal eye infections [111]. Bhageerathy and Prasanth (2024) furthered this research by demonstrating that a prolonged release cubogel formulation of moxifloxacin hydrochloride could provide sustained drug release and increased bioavailability, potentially improving bacterial conjunctivitis therapy [112].

The enormous body of research that has been conducted over the course of more than a decade highlights the revolutionary potential of drug nanoparticles based on LCs in the context of ocular drug delivery. The aforementioned developments shed light on the capability of licensed clinical practitioners (LCNPs) to boost patient compliance, reduce the frequency of doses, and improve therapeutic outcomes, thereby paving the way for future innovations in ocular pharmacotherapy. In order to fully grasp the benefits of these advanced delivery methods in treating a wide variety of ocular disorders, it will be necessary to conduct additional research and clinical validation as the area continues to undergo development. Table 5 provides a summary of LCNPs utilized in ocular drug delivery.

While traditional LLC applications have focused predominantly on their structural versatility and controlled drug release properties, recent advancements have significantly broadened the scope of their utility.

## 6. Beyond Traditional Lyotropic Techniques

The field of LLCs has witnessed remarkable advancements that go beyond traditional techniques. These developments span predictive modeling, an automated analysis, and novel applications, significantly broadening the scope of research and application of LLCs.

### 6.1. Predictive Modeling and Molecular Ordering

Advances in predictive modeling have significantly impacted the study of LLC, particularly through the use of machine learning (ML) techniques [18]. For instance, artificial neural networks (ANNs) have been successfully applied to predict the phase behavior of LLC systems, a crucial factor in designing drug delivery mechanisms [18]. The study by Tu C. Le and Nhiem Tran applies ML, particularly ANNs, to predict the self-assembled nanostructures of LLCs formed by monoolein (MO) and phytantriol (PHYT) as functions of temperature and fatty acid additives. The researchers prepared LLC nanoparticles by dissolving MO, PHYT, and fatty acids in ethanol, followed by solvent evaporation and sonication [18]. The resulting LLC structures were characterized using SAXS to identify specific phases based on scattering profiles, such as inverse hexagonal (HII) and bicontinuous cubic phases. ANNs were employed to simulate and predict the formation of these LLC phases under various conditions, reducing the need for extensive experimental trials [18]. The models showed high accuracy, ranging from 70% to 100% across different mesophases, demonstrating the potential of ML in optimizing LLC-based drug delivery systems [18].

In addition to phase behavior, molecular ordering within LLCs is another critical factor in their application, particularly in fields such as optical materials and nanotechnology. The ML has been utilized to predict and control the molecular ordering of LLCs, which directly influences their optical properties and functionality [19]. For example, precise molecular alignment is vital in creating materials with specific refractive indices or other optical characteristics, making this predictive capability invaluable for developing advanced optical devices [19]. The study by Inokuchi et al., investigates the use of ML to predict molecular ordering and phase transition temperatures in binary LC systems, which are mixtures of two types of LC molecules [19]. The research utilized dissipative particle dynamics (DPD) simulations to model these LC molecules’ self-assembly and phase transitions under varying concentrations and temperatures. The ML models, particularly Random Forest regression, were developed using the simulation data to predict the order parameter and phase transition temperatures of the binary LC systems [19]. The results demonstrated high prediction accuracy, confirming that ML can effectively simulate the complex behaviors of polydisperse LC systems, potentially accelerating material design and reducing reliance on trial-and-error experimentation.

Moreover, ML techniques have also been employed to estimate physical properties from the textures of LLCs. By analyzing images of LLC textures, ML models can predict properties such as the average order parameter and cholesteric pitch length with high precision, providing a non-invasive method for assessing the physical characteristics of LLCs [118]. The study by Sigaki et al. presents a novel approach that integrates physics-inspired image quantifiers—permutation entropy and statistical complexity—with ML techniques to extract the physical properties of nematic and cholesteric liquid crystals directly from texture images [118]. While traditional imaging techniques have been widely used to study liquid crystals, direct extraction of physical properties from texture images remains underexplored [118]. Using both simulated and experimental texture data, the proposed method accurately predicts critical physical properties, such as average order parameters, sample temperature, and cholesteric pitch length [118]. This approach demonstrates high predictive precision and potential for broader application in more complex liquid crystal experiments and in analyzing other materials studied through imaging techniques. As ML continues to advance, ethical considerations, such as ensuring unbiased data use [119] and maintaining data privacy [120,121], remain pivotal to the responsible application of these technologies [122].

Also, the study by Dierking et al. explores the use of various supervised machine learning architectures, specifically convolutional neural networks (CNNs) and incep-tion networks, for classifying liquid crystal phases, including isotropic, nematic, cholesteric, and smectic phases, from texture images [123]. The researchers tested different model architectures, varying the number of layers, inception blocks, and regularization techniques such as dropout layers and image flipping to enhance classification accuracy [123]. The inception network with two blocks generally achieved the best results, demonstrating that model complexity should match the classification task to avoid overfitting [123]. The study highlights the potential of machine learning as a valuable tool for automating phase identification in liquid crystals, with implications for sensor applications and material characterization.

Shan, X., et al. developed an injectable and sprayable LLC platform specifically designed to treat hormone-sensitive and castration-resistant prostate cancer [124]. The goal was to deliver drugs directly to tumor sites, minimizing side effects. The study employed optimization algorithms, including deep learning models, to fine-tune the LLC composition for enhanced drug delivery. To maximize treatment efficacy, neural networks were used to predict the best formulation parameters, such as drug release rates and stability under physiological conditions [124].

The study of Araz, O.U., et al. focused on developing a hybrid sensing system that used LLCs to detect toxic gases, which are often found in healthcare environments or industrial settings [125]. Adaptive Neuro-Fuzzy Inference Systems (ANFIS) were used to enhance the accuracy and sensitivity of the LLC-based sensors [125]. ANFIS combines neural networks with fuzzy logic, allowing the system to learn and adapt to new data patterns over time, making it highly effective in predicting gas concentrations and types from sensor outputs.

### 6.2. Enhanced Biosensing Capabilities

The application of AI and machine learning to LLC-based biosensors has led to significant improvements in their sensitivity and specificity [21]. Biosensors leveraging LLCs are particularly effective in detecting biological molecules, pathogens, and biomarkers due to the unique optical properties of these LCs [126,127]. AI-driven algorithms have been employed to analyze the optical responses of LLCs when exposed to various biological targets, enabling more accurate and rapid detection.

One such advancement is the use of convolutional neural networks (CNNs) to process and classify the optical images generated by LLC biosensors. These networks can detect subtle changes in the optical patterns that indicate the presence of specific pathogens or biomarkers, significantly improving the sensor’s performance [21]. This approach is crucial in medical diagnostics, environmental monitoring, and food safety, where accurate and timely detection is critical [21].

Moreover, the use of AI in optimizing LLC biosensors has expanded their applications in detecting a broader range of substances, enhancing their utility in various fields. For example, researchers have demonstrated the capability of LLC-based sensors to detect entire microorganisms, which can be vital in detecting bacterial contamination in water or food sources [126].

### 6.3. Potential Synergies between Lyotropic Liquid Crystals and Deep Eutectic Solvents

Recent advancements in material science have revealed the potential of Deep Eutectic Solvents (DES) as versatile solvents that can significantly influence the self-assembly, stability, and phase behavior of lyotropic liquid crystals (LLCs) [128]. DES are recognized for their unique physicochemical properties, such as low volatility [129,130], tunable polarity [131,132], and biocompatibility [133,134], which make them ideal candidates for applications in green chemistry [129,135,136], drug delivery [137,138,139], and biotechnology [140]. Incorporating DES into LLC systems can modify the interactions between surfactants and other amphiphilic molecules, leading to novel mesophase structures that are otherwise challenging to achieve with traditional aqueous or organic solvents [141].

One key area of synergy is the enhancement of the solubilization and stabilization of bioactive compounds within LLC matrices when DES are employed as co-solvents [142]. This unique solvent environment provided by DES can facilitate the formation of more complex LLC phases, such as bicontinuous cubic or lamellar structures, which are critical for advanced applications like drug delivery, enzyme immobilization, and biosensing [143]. Furthermore, DES have been shown to impact the rheological properties and thermal stability of LLC systems [128], thus broadening the scope of their practical use in various industrial and pharmaceutical settings.

Integrating DES into LLCs enhances their functional properties and aligns with the growing demand for sustainable and environmentally friendly solvents. By modifying the polarity and hydrogen-bonding capabilities of the solvent environment, DES can fine-tune the self-assembly processes within LLCs, enabling the design of more efficient and responsive materials [144]. This promising interplay between DES and LLCs opens new avenues for research and development, particularly in the fields of green chemistry, smart materials, and personalized medicine.

### 6.4. Automated and Real-Time Analysis

An automated analysis using deep learning, particularly CNNs, has revolutionized how data from lyotropic LC-based sensors are processed. Traditionally, analyzing optical data from these sensors required manual interpretation, which was time-consuming and prone to human error. However, deep learning models have automated this process, significantly improving both the speed and accuracy of detection.

In practical terms, CNNs are trained on large datasets of optical images to recognize and classify different patterns corresponding to the presence of various biological molecules or environmental changes [20]. This automation is particularly valuable in real-time applications, such as continuous monitoring systems in healthcare or environmental sensing, where quick and accurate detection is essential [20].

Furthermore, ML techniques have been employed to monitor the structural evolution of LLCs in real-time under dynamic conditions, such as shear flow or temperature variations [145]. By analyzing how LLC structures change over time, these models provide insights into the material’s behavior during processing, allowing researchers to optimize conditions for desired outcomes. This real-time monitoring is crucial for applications in materials science and engineering, where maintaining specific structural properties during processing can significantly impact the final product’s performance [145].

### 6.5. Designing Light-Responsive Systems

Significant advances have been made in the design of light-responsive LLCs systems. These systems act as molecular switches that can be triggered by light to control the release of drugs or other molecules. The ability to use light as a non-invasive and precise trigger is particularly beneficial in therapeutic applications, where controlled release is critical for treatment efficacy [146].

Researchers have developed light-responsive LLC systems that incorporate photoactive units, such as azobenzene derivatives, into the liquid crystal matrix [146]. Upon exposure to light, these units undergo a conformational change, altering the LLC’s structure and triggering the release of encapsulated molecules. This method provides exquisite control over the timing, dosage, and spatial distribution of the release, making it an ideal candidate for applications such as targeted drug delivery or responsive materials [146].

### 6.6. Exploring Nonlinear Optical Properties

In the realm of optical materials, LLCs have also been explored for their nonlinear optical properties, particularly in DNA-based LLCs, which represents a crucial advancement in the development of materials with tailored optical responses, which are highly sought after in fields such as biophotonics and telecommunications [147,148,149].

The nonlinear optical properties of DNA-based LLCs stem from their unique molecular structure, which can be manipulated to achieve specific optical effects, such as second-harmonic generation or optical limiting [150]. These properties are essential for applications in advanced optical devices, where precise control over light-matter interactions is required. By understanding and harnessing these nonlinear optical behaviors, researchers can develop new materials with applications in photonic circuits, sensors, and other optical technologies [150].

## 7. Conclusions

This review highlights the transformative impact of lyotropic liquid crystals (LLCs) in ocular drug delivery by enhancing bioavailability, prolonging retention times, and enabling controlled release, thereby overcoming the limitations of traditional ophthalmic formulations. We provide foundational insights into LLCs, including their classification, formation, and critical-packing parameter (CPP) role in mesophase structure determination. Expanding beyond ocular applications, we highlight LLCs’ effects on other physiological systems and explore the integration of machine learning and AI-driven predictive modeling to optimize LLC-based formulations. This innovative approach accelerates development, reduces experimental dependency, and broadens LLC applications in biosensing and diagnostics. Combining fundamental LLC knowledge with computational techniques is essential for advancing targeted, patient-friendly ocular therapies.

## Figures and Tables

**Table 1:** Ocular Diseases and Challenges.

Segment	Disease	Challenges in Drug Delivery	References
Anterior Segment	Dry Eye Syndrome	High tear turnover leads to rapid drug clearance and low corneal penetration.	[5]
Anterior Segment	Glaucoma	Need for sustained IOP control; low bioavailability of topical agents; frequent dosing required.	[6]
Anterior Segment	Allergic Conjunctivitis	Requires rapid action with minimal systemic absorption; barriers like the conjunctival epithelium.	[7]
Anterior Segment	Anterior Uveitis	High risk of systemic side effects with oral therapy; low penetration with topical steroids.	[8]
Anterior Segment	Cataract	Requires precise drug delivery post-surgery to prevent infection and inflammation.	[9]
Posterior Segment	Age-Related Macular Degeneration (AMD)	Poor penetration of drugs to the retina; risk of damage with repeated injections.	[10]
Posterior Segment	Diabetic Retinopathy (DR)	Sustained delivery needed to reduce frequent IVT injections; blood–retinal barrier limits drug access.	[11]
Posterior Segment	Diabetic Macular Edema (DME)	Requires precise targeting to avoid damage to the retina; high systemic absorption risks with systemic therapy.	[10]
Posterior Segment	Proliferative Vitreoretinopathy	High recurrence rate necessitates long-term therapy; limited options for effective drug penetration.	[12]
Posterior Segment	Cytomegalovirus Retinitis	Often requires systemic treatment with significant side effects; local delivery challenging due to retinal barriers.	[13]
Posterior Segment	Retinal Vein Occlusion	Requires sustained anti-VEGF therapy; risks associated with repeated intraocular injections.	[14]

**Table 2:** Ocular Administration Routes.

Route	Application	Pros	Cons	References
Topical (Eye Drops)	Dry Eye Syndrome, Glaucoma, Conjunctivitis	Easy administration; high patient compliance; direct application to the eye.	Low bioavailability (<5%); frequent dosing required; low posterior segment delivery.	[23]
Systemic	Severe Infections, CMV Retinitis, Uveitis	Bypasses ocular barriers; effective for widespread or severe infections.	Low ocular bioavailability; significant systemic side effects; poor targeting.	[24]
Intraocular (IVT)	AMD, DR, Retinal Vein Occlusion, DME	Direct delivery to posterior segment; high drug concentration at target site.	Invasive; risks of endophthalmitis, retinal detachment, and cataracts; repeated injections needed.	[25]
Periocular	Posterior Uveitis, DR, DME	Direct targeting of posterior segment; reduces systemic exposure.	Risk of local complications; requires technical expertise; may cause discomfort.	[26]
Intracameral (IC)	Post-Cataract Surgery	High local drug concentration; effective prevention of post-surgical infections.	Invasive; risks include increased IOP, corneal edema, or endothelial cell loss.	[27]
Subconjunctival (SC)	Posterior Uveitis, DME, DR	Enhanced drug penetration to the posterior segment; reduced dosing frequency.	Potential for local irritation or fibrosis; requires careful injection technique.	[28]
Retrobulbar (RB)/Peribulbar (PB)	Anesthesia for Cataract or Retinal Surgery	Effective anesthesia for surgical procedures; long-lasting anesthetic effects.	Risks include optic nerve injury, globe perforation, or hematoma formation.	[29]
Sub-Tenon (ST)	Uveitis, DME, Surgical Anesthesia	Safer than RB/PB injections; avoids sharp needles; effective for anti-inflammatory delivery.	Less effective for deep retinal conditions; potential for discomfort and patient anxiety.	[30]

**Table 3:** Summary of Innovative Ocular Drug Delivery Systems and Nanoparticle-Based Approaches.

System Type	Mechanism	Applications	Benefits	Limitations	Reference
Contact Lenses	Sustained drug release through the cornea	Glaucoma	Prolonged contact with corneal tissues increases bioavailability and reduces dosing frequency.	Limitation to certain drug types may require patient adaptation.	[44]
Punctum Plugs	Inhibit tear drainage; sustained drug release	Dry Eye Syndrome	Prolonged drug retention on the ocular surface reduces the need for frequent dosing.	May cause discomfort; potential for blockage or infection.	[45]
Ocular Implants	Long-term, controlled drug release at targeted sites	Uveitis	Provides consistent therapeutic levels; minimizes systemic exposure; long-lasting effects.	Invasive procedure; risk of local complications; potential for device migration.	[46]
Microneedles	Minimally invasive drug delivery through ocular barriers	AMD, Retinal Disorders	Direct drug delivery to the retina or vitreous reduces systemic exposure and is less invasive than IVT.	Requires skilled application; potential for local irritation or damage.	[47]
In Situ Gels	Liquid-to-gel transformation upon contact with ocular fluids	Glaucoma, Uveitis	Prolonged drug release; improved patient compliance; reduced dosing frequency.	Limited to specific formulations; potential for discomfort during gel formation.	[48]
Ocular Inserts	Controlled drug release from solid or semi-solid inserts	Glaucoma, Dry Eye Syndrome	Sustained drug release; improves patient adherence; reduces systemic side effects.	It may cause discomfort or foreign body sensation, and it has the potential for dislodgement.	[49]
Nanomicelles	Self-assembly of amphiphilic block copolymers in aqueous environments	Glaucoma, Uveitis	Enhanced solubility of hydrophobic drugs; improved corneal penetration; sustained release.	Limited size capacity for drug encapsulation; potential for polymer-related toxicity.	[50]
Polymeric Nanoparticles	Biodegradable polymer-based nanocarriers	AMD, Glaucoma, Ocular Infections	Prolonged drug retention; biocompatible and biodegradable; potential for targeted delivery.	Potential immunogenicity; complex manufacturing process; stability issues.	[51]
Lipid-Based Nanoparticles	Solid and liquid lipids	Post-Surgical Inflammation, Retinal Disorders	Improved drug loading, sustained release profiles, and better patient tolerance.	Risk of lipid crystallization affecting drug release; challenges in large-scale production.	[52]
Cubosomes	Nanostructured particles with cubic symmetry	Uveitis, Retinal Disorders	Efficient encapsulation of both hydrophilic and hydrophobic drugs; enhanced stability.	Opaque appearance might limit visual applications; complex formulation process.	[53]
Dendrimers	Hyperbranched polymer-based carriers	Anti-VEGF Therapy	High surface area for drug conjugation; controlled release.	Expensive to produce; potential toxicity due to surface charge; complex synthesis.	[54]
Nanowafers	Dissolvable wafer that releases drugs over time	Ocular Infections	Sustained release; enhances therapeutic efficacy; reduces systemic exposure.	Limited by the types of drugs that can be loaded; potential for irritation.	[55]

**Table 4:** Summary of bulk-forming LLCs used in ocular drug delivery, highlighting the drug, carrier system, amphiphilic lipid, and significant outcomes.

Drug	Carrier System	Amphiphilic Lipid	Significant Outcome	Reference
Vancomycin HCl	Bulk hexagonal and cubic phases	GMO	GMO-based liquid crystalline phases were able to increase the bioavailability and effectiveness of vancomycin in the eye.	[68]
Vancomycin HCl	Bulk LC phases modulated with tuning agents	GMO	Effectively delivering Vancomycin HCl in vivo intravitreally for 2880 min.	[82]
Pilocarpine Nitrate	Reversed bicontinuous cubic (QII) phase	PHY	Pilocarpine nitrate could maintain sustained release from the gels for 12 h.	[83]
Pilocarpine Nitrate	Cubic (Q2) and hexagonal (H2) phases	PHY	LC gels exhibited sustained release behavior for pilocarpine nitrate and more cumulative drug penetration across the cornea.	[71]
Dexamethasone	ISLG	PHY	Significant enhancement in corneal penetration.	[84]
Resveratrol	ROLG	GMO	ROLGs demonstrated strong retention on the ocular surface and a high capacity for drug loading.	[66]
Acyclovir	The lamellar phase transitions into a cubic phase in situ	GMO or PHY	The enhanced bio-adhesion and extended residence time of the LC systems led to improved ocular drug bioavailability.	[85]

**Table 5:** Summary of LCNPs used in ocular drug delivery, highlighting the drug, carrier system, amphiphilic lipid, stabilizer, and significant outcomes.

Drug	Carrier System	Amphiphilic Lipid	Stabilizer	Significant Outcome	Reference
Dexamethasone	Cubosome	GMO	Poloxamer 407	Significant improvement in dexamethasone ocular bioavailability	[15]
Pilocarpine nitrate	Cubosome	GMO	Poloxamer 407	Improved bioavailability superior to commercial eye drops	[87]
Pirfenidone	Cubosome	GMO	Poloxamer 407	Sustained release profile compared to drug solution	[94]
Cyclosporine A	Cubosome	GMO	Poloxamer 407	Enhanced penetration and retention compared to oil solution	[81]
Tropicamide	Cubosome	GMO	Poloxamer 407	Faster onset and higher intensity of mydriatic action than conventional ophthalmic solution	[91]
Brinzolamide	Cubosome	GMO	Poloxamer 407	Prolonged drug release compared to commercial product	[84]
Riboflavin	Cubosome	Peceol^®^	Poloxamer 407	Improved preocular retention and ocular bioavailability	[88]
D-Mannitol	Cubosome	GMO	Poloxamer 407	Effective as potential carriers for improved ocular delivery	[96]
Tetrandrine	Cubosome	GMO	Poloxamer 407, Gelucire 44/14	Prolonged release profile compared to drug solution	[90]
Tobramycin	Cubosome	GMO	Poloxamer 407	Improved effectiveness over marketed tobramycin eye drops	[97]
Bromfenac	Cubosome	GMO	Poloxamer 407	Longer duration of action and higher bioavailability than drug solution	[101]
Acetazolamide	Cubosome	GMO	Poloxamer 407, Transcutol P	Greater therapeutic efficacy than commercial products	[113]
Brimonidine	Cubosome	GMO	Poloxamer 407	Sustained IOP-lowering effect for 17.6 h, compared to 1.9 h with Alphagan^®^P	[95]
Ketorolac	Cubosome	Peceol^®^	Poloxamer 407	Significantly increased transcorneal penetration	[92]
Flurpiprofen	Cubosome	GMO	Poloxamer 407	Enhanced transcorneal permeation	[114]
Beclomethasone	Cubosome	GMO	Poloxamer 407	Increased bioavailability and improved ocular permeability	[115]
Vancomycin	Cubosome	GMO	Poloxamer 407	Considerable decrease in severity of keratitis	[97]
LM22A-4	Cubosome	PHY	Pluronic 127	Successfully targeted posterior retina and optic nerve head in vivo	[116]
Latanoprost	Cubosome	PHY	Poloxamer 407	Persisted IOP reduction for at least 9 days, compared to 24 h with commercial formulation	[99]
Sertaconazole	Cubosome	GMO	Poloxamer 407, Poloxamer 188	Excellent in vivo corneal absorption and tolerability	[93]
Fluconazole	Cubosome	GMO	Poloxamer 407	More effective and safer for treating keratomycosis than aqueous drug solution	[102]
Travoprost	Cubosome	GMO	Poloxamer 407, Tween^®^80	Decrease in intraocular pressure lasting 48–72 h compared to commercial formulation	[103]
Ketoconazole	Cubosome	GMO	Poloxamer 407	Boosted antifungal activity in rabbit-induced fungal keratitis	[100]
Loteprednol etabonate	Cubosome	Lipoid S 75	Poloxamer 407, Poloxamer 338, Transcutol P	Improved ocular retention, efficacy, and patient compliance	[104]
Fluorometholone	Cubosome	GMO	Poloxamer 407	Sustained release and increased permeability	[105]
Triamcinolone	Cubosome	GMO	Poloxamer 407	Superior drug delivery and therapeutic outcomes	[106]
Luteolin	Cubosome	GMO	Poloxamer 407	Remarkable efficacy in reducing intraocular pressure and inflammation	[107]
Gemifloxacin mesylate	Cubosome	GMO	Poloxamer 407	Greater potency, significant reductions in corneal opacity and inflammation	[117]
Moxifloxacin hydrochloride	Cubosome	GMO	Poloxamer 407	Sustained drug release and increased bioavailability	[108]
Fluorometholone	Cubosome	GMO	Poloxamer 407	Improved ocular bioavailability and drug release	[109]
Acetazolamide	Cubosome	GMO	Poloxamer 407, Polyvinyl alcohol	Increased corneal penetration and extended drug release	[110]
Fenticonazole nitrate	Cubosome	GMO	Poloxamer 188, Poloxamer 407	Enhanced corneal absorption and permeation	[111]
Moxifloxacin hydrochloride	Cubosome	GMO	Poloxamer 407	Increased permeability and sustained drug release	[112]

## Data Availability

Data is contained within the article.
